# Immune Evasion, a Potential Mechanism of Trichothecenes: New Insights into Negative Immune Regulations

**DOI:** 10.3390/ijms19113307

**Published:** 2018-10-24

**Authors:** Qinghua Wu, Wenda Wu, Tanos C. C. Franca, Vesna Jacevic, Xu Wang, Kamil Kuca

**Affiliations:** 1 College of Life Science, Yangtze University, Jinzhou 434025, China; wqh212@hotmail.com; 2 College of Veterinary Medicine, Nanjing Agricultural University, Nanjing 210095, China; 3 Laboratory of Molecular Modeling Applied to the Chemical and Biological Defense, Military Institute of Engineering, Praça General Tiburcio 80, Rio de Janeiro 22290-270, RJ, Brazil; tanosfranca@gmail.com; 4 Center for Basic and Applied Research, Faculty of Informatics and Management, University of Hradec Kralove, Rokitanskeho 62, 500 03 Hradec Kralove, Czech Republic; 5 Department of Experimental Toxicology and Pharmacology, National Poison Control Center, Military Medical Academy, 11 Crnotravska St, 11 000 Belgrade, Republic of Serbia; v_jacevic@yahoo.com; 6 Medical Faculty of the Military Medical Academy, University of Defense in Belgrade, 1 Pavla Jurišića-Šturma St, Belgrade 11000, Republic of Serbia; 7 Department of Chemistry, Faculty of Science, University of Hradec Kralove, 500 03 Hradec Kralove, Czech Republic; 8 National Reference Laboratory of Veterinary Drug Residues (HZAU) and MAO Key Laboratory for Detection of Veterinary Drug Residues, Huazhong Agricultural University, Wuhan 430070, China

**Keywords:** immune evasion, trichothecenes, T-2 toxin, immunotoxicity, negative immune regulations

## Abstract

Days ago, the Nobel Prize in Physiology or Medicine 2018 was awarded jointly to James P. Allison and Tasuku Honjo “for their discovery of cancer therapy by inhibition of negative immune regulation”. This news has increased the attention on immunotoxicity and immune evasion mechanisms, which are once again hot research topics. Actually, increasing lines of evidence show that trichothecene mycotoxins have a strong immunosuppressive effect. These mycotoxins suppress the host immunity and make them more sensitive to the infection of pathogens, including bacteria and viruses. However, the underlying mechanism(s) in this context is still poorly understood. Interestingly, recent work showed that an immune evasion mechanism might be involved in trichothecene immunotoxicity. In this work, we discuss the potential immune evasion mechanism in trichothecene immunotoxicity. More importantly, under these circumstances, we are pleased to compile a Special Issue entitled “Biochemistry, Molecular Biology, and Toxicology of Natural and Synthetic Toxins” for the *International Journal of Molecular Sciences* (*IJMS*). Researchers are encouraged to share their latest interesting findings with the readers of *IJMS*.

Mycotoxins are unavoidable contaminants in the nature. Today, many countries are working hard to control mycotoxin contaminants. Trichothecenes are a large group of chemically related mycotoxins, mainly produced by the fungi of the *Fusarium* genus [1]. They initiate a wide range of toxic effects, including emesis, diarrhoea, and haemorrhaging. Moreover, trichothecenes are highly immunotoxic, and thus can break the host immune functions [2]. In recent years, the frequent global occurrence of livestock diseases has acted as a bottleneck, restricting the sustainable development of animal husbandry. Mycotoxin contamination in feed is an important risk factor for animal susceptibility to pathogens and diseases [3].

Currently, the mechanism(s) underlying trichothecene immunotoxicity is not fully understood. Studies have shown that oxidative stress is a crucial toxic effect of trichothecenes that causes DNA cleavage and apoptosis [4]. These toxins activate a rapid production of reactive oxygen species (ROS), increase lipid peroxidation, induce oxidative stress, and cause apoptosis. The Pestka group at Michigan State University focused on the topic of trichothecene immunotoxicity for nearly 30 years. They showed that trichothecenes cause ribosomal stress response and activate a series of signalling pathways such as NF-κB, MAPK, and JAK/STAT. Trichothecenes also regulate apoptosis-related signal molecules such as IL-6, IL-1β, and TNF-α. MAPK phosphorylation can be activated after these toxins bind to the peptidyl of ribosomes to regulate immune responses and apoptosis [5,6].

As discussed in our previous work [2], trichothecenes have both negative and positive immune regulations. How to reduce the immunosuppressive effect of mycotoxins, or how to transfer the immunosuppressive effect to the active immune effect, thereby increasing the host resistance to environmental pathogens, is an urgent problem. In October, the Nobel Prize in Physiology or Medicine 2018 was jointly awarded to James P. Allison and Tasuku Honjo for their discovery of cancer therapy by the inhibition of negative immune regulation [7]. Allison and Honjo uncovered a new way to inhibit negative immune molecular programmed cell death 1 (PD-1). PD-1 blocking has proven to be more effective, especially in the treatment of lung cancer, kidney cancer, and melanoma. The PD-1/PD-Ligand 1(PD-L1) pathway is considered the primary mechanism by which tumours evade immune system elimination [8]. Intriguingly, recent studies further revealed that an “immune evasion” mechanism is involved in trichothecene immunotoxicity, through which the toxins can escape host resistance and immune repair [2]. Normally, the term “immune evasion” refers to parasites, viruses, and tumour cells that escape the host immune defences through mechanisms antigenic disguise, surrounding environment modulation, and inhibiting or destroying the host immune response [2,9]. During the immune evasion effect, some typical immune genes including *Interferon-γ* (*IFN)-γ*, *transforming growth factor-β* (*TGF-β*), and *toll-like receptors* (*TLR*) are downregulated by bacteria, parasites, or tumours [10] Much evidence has shown that trichothecenes significantly downregulate *IFN-γ* expression in pigs and rats, thereby reducing the host resistance to viruses and repairing ability [11,12]. For example, T-2 toxin suppresses *IFN-γ* expression, which is caused by reovirus infection. Type B trichothecenes deoxynivalenol (DON) reduces *IFN-γ* gene expression in pigs and reduces their immune response to vaccine ovalbumin. DON also reduces *IFN-β* expression and promotes the cell apoptosis [13]. Recently, we found that T-2 toxin reduces the mRNA expression of *TLR* in RAW264.7 cells, which is consistent with the results of earlier studies reporting that DON inhibits the TLR-MyD88 signal [14]. As is known, *TLR* is a target gene for immune evasion in tumour cells. Thus, trichothecenes may shape the immune system to allow pathogens to escape the host immune response [2]. We further suspect that the immune evasion mechanism may have a relationship with the autophagy process, since many studies showed that trichothecenes activate autophagy and protects against apoptosis. However, this hypothesis remains to be tested. Studies that explore the immune evasion mechanisms of trichothecenes are warranted.

At present, some urgent problems should be solved in the field of trichothecene immunotoxicity. For example, what are the upstream regulatory targets for trichothecene immunotoxicity? It is known that trichothecenes can cause both immune suppressive and immune active effects, but what are the molecular mechanisms that underlie their immune balance? Based on the above mechanisms, how can trichothecene immunotoxicity be prevented and controlled? In nature, animals are often exposed to a variety of mycotoxins. Therefore, how the animal immune response be regulated under the combined action of various mycotoxins? These problems are also key issues for future studies of the immunotoxicity of mycotoxins and toxins. Under these circumstances, we are pleased to have this opportunity to compile a Special Issue entitled “Biochemistry, Molecular Biology, and Toxicology of Natural and Synthetic Toxins” for the *International Journal of Molecular Sciences* (*IJMS*). This Special Issue belongs to the section of “Molecular Toxicology” and will focus on both synthetic and naturally-occurring toxins (including mycotoxins), as well as their chemistry and biochemistry. Their effects will be solved at the molecular level. Finally, their toxic potential and possible antidotes used in case of intoxications will be thoroughly discussed. We welcome researchers to submit their relevant research articles and excellent work on toxins (and mycotoxins) to this Special Issue, with the aim of sharing the latest ideas and progresses in this field with the readers of *IJMS*.
